# High-level Aminoglycoside Resistance and Reduced Susceptibility to Vancomycin in Nosocomial Enterococci

**DOI:** 10.4103/0974-777X.68534

**Published:** 2010

**Authors:** Luna Adhikari

**Affiliations:** *Department of Microbiology, Sikkim-Manipal Institute of Medical Sciences (SMIMS) and Central Referral Hospital (CRH), 5th Mile Tadong, Gangtok, Sikkim, India- 737 102*

**Keywords:** High-level aminoglycoside resistance, Nosocomial enterococci, Reduced susceptibility to vancomycin

## Abstract

**Objectives:**

The objectives of the present study were to identify the species of enterococci isolated from nosocomial infections and to determine the antibiotic susceptibility pattern with reference to high-level aminoglycosides and vancomycin.

**Materials and Methods:**

Enterococci were isolated from various clinical samples collected from patients after 72 hours of hospitalization. Various species of *Enterococcus* were identified by standard methods. High-level aminoglycoside resistance and vancomycin susceptibility in enterococci were detected by disk-diffusion and agar-screen methods.

**Results:**

One hundred eighty enterococcal strains were isolated from various clinical samples. Various species of *Enterococcus — Enterococcus fecalis* 130 (72.22%), *Enterococcus casseliflavus* 24 (13.33%), *Enterococcus fecium* 17 (9.44%), *Enterococcus durans* 7 (3.89%) and *Enterococcus dispar* 2 (1.11%) — were isolated. The highest resistance to aminoglycoside was observed among *E. fecium*, followed by *E. durans, E. fecalis* and *E. casseliflavus*, both by disk-diffusion and agar-screen methods. The high-level aminoglycoside resistance (HLAR) was significantly (*P*<0.05) higher in *E. fecium* by agar-screen method. All enterococci showed minimum inhibitory concentration (MIC) of ≤8 μg/mL to vancomycin. Sixteen (12.31%) *E. fecalis* and 3 (12.5%) *E. fecium* strains were intermediately resistant to vancomycin (MIC= 8 μg/mL), whereas other strains were susceptible to vancomycin.

**Conclusion:**

The occurrence of high-level aminoglycoside resistance in enterococcal isolates in our setup was high. Even though none of the enterococcal strains showed resistance to vancomycin, yet reduced susceptibility to vancomycin was noticed in our study. This would require routine testing of enterococcal isolates for HLAR and vancomycin susceptibility. Agar-screen method was found to be superior to disk-diffusion method in detecting resistant strains to aminoglycosides and vancomycin.

## INTRODUCTION

Enterococci, though commensal in adult feces, are important nosocomial pathogens.[1–3] *E. fecalis* is the most common cause (80%-90%) of infection, followed by *E. fecium* (10%-15%).[4] Their emergence in the past two decades is in many respects attributable to their resistance to many commonly used antimicrobial agents (aminoglycosides, cephalosporins, aztreonam, semisynthetic penicillin, trimethoprim-sulphamethoxazole)[5,6] and ease with which they appear to attain and transfer resistant genes,[7] thus giving rise to enterococci with high-level aminoglycoside resistance (HLAR) and glycopeptide resistance.

A common regime for treatment of serious enterococcal infections is the combination of cell-wall inhibitors, such as penicillin, ampicillin or vancomycin; with aminoglycosides, such as streptomycin or gentamicin.[8] The addition of cell-wall inhibitor agent helps in the penetration of the aminoglycoside into the bacterial cytoplasm, making the intrinsically resistant organism aminoglycoside sensitive. Reduced susceptibility to vancomycin will interfere with the penetration of the aminoglycoside into the bacterial cytoplasm, thus making the synergism ineffective. The presence of HLAR in enterococci, defined as minimum inhibitory concentration of ≥2000 μg/mL of aminoglycoside for the isolate, makes the synergism of cell-wall inhibitor and aminoglycoside ineffective.[9] The main objectives of the present study were to identify the species of enterococci isolated from nosocomial infections and to determine the antibiotic susceptibility pattern with reference to high-level aminoglycosides and vancomycin.

## MATERIALS AND METHODS

### Study population

The study population included patients of all age groups hospitalized at Government Wenlock Hospital, Government Lady Goschen Hospital, Kasturba Medical College Hospital, Attavar; and University Medical Centre, Mangalore, Karnataka, India. Infection was considered nosocomial if it developed more than 72 hours after admission to hospital.[10]

### Isolation and identification

Enterococci were isolated from various clinical samples (pus, urine, blood and peritoneal aspirate)

Enterococci were identified using standard methods based on gram staining, catalase reaction, bile aesculin, growth in 6.5% NaCl and sugar-fermentation reactions.[4,11]

### Antibiotic susceptibility of *Enterococcus* species

Antibiotic sensitivity testing of enterococci was performed using Kirby-Bauer disk-diffusion method.[12] Mueller-Hinton agar supplemented with 5% sheep blood was used. The antibiotic disks were purchased from Hi Media, Mumbai. The antibiotic disks and their potency were as follows: ampicillin (10 μg), gentamicin (120 μg), penicillin (10 U), streptomycin (300 μg) and vancomycin (30 μg).The controls were *S. aureus* ATCC 25923 and *E. fecalis* ATCC 29212.

### Detection of HLAR in enterococci by disk-diffusion and agar-dilution methods

HLAR in enterococci was detected by disk-diffusion method and agar-screening method.[13] In disk-diffusion method, isolated colonies of enterococci were inoculated into peptone water to get bacterial suspension that was equivalent to McFarland 0.5 standard. Lawn culture on blood agar was done by swabbing the bacterial suspension. High-level (120 μg) gentamicin and streptomycin (300 μg) disks were placed on the agar medium. Plates were incubated at 37°C for 24 hours, and diameter of zone of inhibition was measured. Resistance was indicated by no zone; and susceptibility, by a zone of diameter ≥10mm. Strains with inhibition zones of 7 to 9 mm were re-tested by dilution method. In agar-screen method, brain-heart infusion agar (BHIA, Hi Media, Mumbai) was supplemented with 500 μg/mL gentamicin and 2000 μg/mL streptomycin separately. The plates were inoculated by spotting 10 μL of bacterial suspension that was equivalent to McFarland 0.5 standard prepared from growth on 24-hour incubated agar plate giving a final inoculum of 10^6^cfu/spot. The plates were incubated at 37°C for 24 hours. Presence of more than one colony or a haze of growth was read as resistance. Aminoglycoside plates which did not show bacterial growth after 24-hour incubation were incubated for additional 24 hours. The test was quality controlled using *E. fecalis* ATCC 29212 (susceptible) and *E. fecalis* ATCC 51299 (resistant)

### Determination of minimum inhibitory concentration of vancomycin.[13]

Agar dilution was used to determine MIC of vancomycin to enterococci. Brain-heart infusion agar (Hi Media, Mumbai) was supplemented with different concentrations of vancomycin. The test organism was grown in broth and the turbidity matched with McFarland 0.5 standard (approximately 1.5 × 10^8^cfu/mL). Spot inoculation of the agar medium was done using 10 μL of bacterial culture. Growth control was used with each series of test. The plates were incubated at 37°C for 24 hours and examined. The minimum concentration of vancomycin which inhibited bacterial growth was considered MIC. Enterococci which had MIC ≥32 μg/mL were considered resistant; MIC of 8-16 μg/mL, as intermediately resistant; and MIC of 4 μg/mL, as susceptible to vancomycin.[14]

### Statistics

Statistical evaluation of the result of antibiotic sensitivity test was done using ‘Z’ test for proportions.

## RESULTS

A total of 180 strains of enterococci were isolated from various clinical samples. One hundred twenty-one (67.22%) strains were isolated from urinary tract infections; 31 (17.22%) strains were from bacteremia, of which 15 (8.33%) strains were from endocarditis, 25 (13.89%) strains were from wound infection and 3 (1.67%) strains were from peritonitis [Table 1]. The male-female ratio was 1.25:1. Various species of *Enterococcus* were isolated — *E. fecalis* 130 (72.22%), *E. casseliflavus* 24 (13.33%), *E. fecium* 17 (9.44%), *E. durans* 7 (3.89%) and *E. dispar* 2 (1.11%)[Table 1]. In disk-diffusion method, of the 130 *E. fecalis*, 32 (24.62%); of the 17 *E. fecium*, 7 (41.18%); of the 24 *E. casseliflavus*, 4 (16.67%); and of the 7 *E. durans*, 3 (42.86%) showed high-level resistance to gentamicin and streptomycin [Table 2]. However, by agar-screen method, 34 (26.15%) *E. fecalis*, 9 (52.94%) *E. fecium*, 4 (16.67%) *E. casseliflavus*, 3 (42.86%) *E. durans* showed high-level resistance to gentamicin and streptomycin [Table 3]. The highest resistance was observed among *E. fecium*, followed by *E. durans*, *E. fecalis* and *E. casseliflavus*, both by disk-diffusion method and agar-screen method. *E. dispar* was sensitive to gentamicin and streptomycin, both by disk-diffusion and agar-screen methods. The HLAR was significantly (*P*<0.05) higher in *E. fecium* by agar-screen method [Table 3]. All the isolates were sensitive to vancomycin by disk-diffusion method [Table 2]. But by agar-dilution method, of the 130 *E. fecalis* isolates, 16 (12.3%); and of the 17 *E. fecium* isolates, 3 (12.5%) had intermediate resistance (MIC= 8 μg/mL) [Table 4]. Fifty-two (28.89%) isolates were resistant to ampicillin and penicillin; of these, 36 (27.69%) isolates were *E. fecalis*, 12 (70.59%) isolates were E. fecium, 2 (8.33%) isolates were *E. casseliflavus* and 2 (28.57%) were *E. durans*. Ampicillin and penicillin resistance was significantly (*P*<0.05) higher in *E. fecium* [Table 2].

**Table 1:** Isolation of *Enterococcus* spp from clinical samples

Clinical samples	Number (%) isolates					
	*E. fecalis*	*E. fecium*	*E. casseliflavus*	*E. durans*	*E. dispar*	Total
Urine	85 (70.25)	10 (8.26)	19 (15.7)	5 (4.13)	2 (1.65)	121
Blood	20 (64.52)	5 (16.13)	4 (12.9)	2 (6.45)	0 (0)	31
Pus						
Surgical wound	12 (48)	2 (8)	1 (4)	0 (0)	0 (0)	15
Nonsurgical wound 05(20)	0 (0)	0 (0)	0 (0)	0 (0)	5	
Burn wound	05 (20)	0 (0)	0 (0)	0 (0)	0 (0)	5
Peritoneal fluid	03 (100)	0 (0)	0 (0)	0 (0)	0 (0)	5
Total	130 (72.22)	17 (9.44)	24 (13.33)	7 (3.89)	2 (1.11)	180

**Table 2:** Antibiotic resistance in enterococci

Antibiotics (disk content)	Number (%) of resistant isolates					
	*E. fecalis (n=130)*	*E. fecium (n=17)*	*E. casseliflavus (n=24)*	*E. durans (n=7)*	*E. dispar (n=2)*
Ampicillin (10 μg)	36 (27.69)	12 (70.59)	2 (8.33)	2 (28.57)	0 (0)
Gentamicin (120 μg)	32 (24.62)	7 (41.18)	4 (16.67)	3 (42.86)	0 (0)
Penicillin (10U)	36 (27.69)	12 (70.59)	2 (8.33)	2 (28.57)	0 (0)
Streptomycin (300 μg)	32 (24.62)	7 (41.18)	4 (16.67)	3 (42.86)	0 (0)
Vancomycin (30 μg)	0 (0)	0 (0)	0 (0)	0 (0)	0 (0)

**Table 3:** High-level aminoglycoside resistance (HLAR) in enterococci by agar-dilution method

Species of *enterococcus*	Number	Number (%) of isolates resistant
*E. fecalis*	130	34 (26.15)
*E. fecium*	17	9 (52.94)
*E. casseliflavus*	24	4 (16.67)
*E. durans*	7	3 (42.86)
*E. dispar*	2	0 (0)
Total	180	50 (27.78)

**Table 4:** Minimum inhibitory concentrations (MICs) of vancomycin to enterococci

Concentration of vancomycin (μg/mL)										
Species of *enterococcus*	0.125	0.25	0.5	1	2	4	8	16	32	64

Number of isolates										
*E. fecalis (n=130)*	0	7	5	15	31	56	16	0	0	0
*E. fecium (n=17)*	0	0	5	1	1	7	3	0	0	0
*E. casseliflavus (n=24)*	0	2	6	5	5	6	0	0	0	0
*E. durans (n=7)*	1	1	2	0	1	2	0	0	0	0
*E. dispar (n=2)*	0	0	0	0	1	1	0	0	0	0
Total (180)	1	10	18	21	39	72	19	0	0	0

## DISCUSSION

Enterococci show intrinsic low-level cross resistance to all aminoglycosides due to decreased uptake of antibiotics.[15] Therefore, there is no meaning in testing susceptibility of clinical isolates of enterococci to low-level aminoglycosides. Enterococci can also exhibit acquired resistance to high level of aminoglycosides. It is very important to know whether the clinical isolate of *Enterococcus* is susceptible to high level of aminoglycosides or not. We used disk-diffusion (using high-potency gentamicin and streptomycin) and agar-screening methods to detect HLAR. Agar-screen method was found superior in identifying HLAR. It is possible that disk-diffusion method may not detect borderline resistance. HLAR was significantly higher among *E. fecium* isolates, an observation which is consistent with that found in previous reports.[16,17] The result of the present study clearly indicates that agar-screen method must be used to confirm HLAR in enterococci. Enterococci are intrinsically resistant to most commonly used antibiotics. Therefore, recommended therapy for serious infections like endocarditis, meningitis or possibly other serious infections in immunodeficient patients includes a cell-wall–active agent such as penicillin or vancomycin, combined with an aminoglycoside like gentamicin or streptomycin. This combination is synergistic in action.[18] However, when an enterococcal strain is resistant to the cell-wall-active agent or has HLAR, there is no synergism and the combination therapy is likely to be unsuccessful. Because of this, it is very important to detect resistance to both the aminoglycosides and the cell-wall-active agents in order to predict the likelihood of synergy. The incidence of infection due to strains of *Enterococcus* with glycopeptides resistance has increased dramatically. It is also important to know that usually these infections occur in a setting where vancomycin is being used. In the present study, all enterococci were found vancomycin susceptible by disk-diffusion method. However, 16 (12.31%) strains of *E. fecalis* and 3 (17.67%) strains of *E. fecium* showed intermediate resistance (MIC, 8 μg/mL) to vancomycin. This observation clearly indicates that disk-diffusion method is not satisfactory to detect vancomycin resistance in enterococci. Clinical laboratories that use disk-diffusion techniques may fail to recognize as resistant those enterococcal strains that have reduced susceptibility to vancomycin. This observation is consistent with that made in a previous report.[19] Early detection of vancomycin resistance in clinically significant *Enterococcus* is important for the management of a case. The treatment of vancomycin-resistant enterococci is a major clinical problem. Vancomycin resistance eliminates the synergistic activity usually achieved by aminoglycoside combination, thus leaving β-lactamase as the only choice to combine with aminoglycosides. However, many of the vancomycin-resistant enterococci are multi-drug resistant. The antibiotic of choice for such multi-drug–resistant enterococci is currently not known.

Drug-resistant enterococci present a challenge for the clinician and the clinical microbiologist because of their increased occurrence in nosocomial infections. The situation obligates the clinical microbiologist to try to identify the most useful active antibiotic for treatment. On the other hand, physicians should use antibiotics appropriately and comply with the infection-control policies in an effort to prevent further spread of these resistant organisms.

### Strength of the study

- The study identified less common species of *Enterococcus — E. casseliflavus, E. durans, E. dispar*.
- The study also found agar-screen method to be superior in identifying HLAR in enterococci.
- The HLAR was found to be significantly higher in *E. fecium* by agar-screen method.
- The study also detected reduced susceptibility to vancomycin in enterococcal strains.

### Limitations of the study

Patients with HLAR and/ or those with reduced susceptibility to vancomycin enterococcal infection could not be followed up, so the outcome of infection with these strains could not be found out.

## CONCLUSION

The occurrence of high-level aminoglycoside resistance in enterococcal isolates in our setup was high. Even-though none of the enterococcal strains showed resistance to vancomycin, yet reduced susceptibility to vancomycin was noticed in our study. This would require routine testing of enterococcal isolates for HLAR and vancomycin susceptibility. Agar-screen method was found to be superior to disk-diffusion method in detecting strains resistant to aminoglycosides and vancomycin.

Recommendations on the basis of this study

The study recommends routine testing of enterococcal isolates for HLAR and vancomycin susceptibility. Agar-screen method should be preferred for detection of HLAR in enterococci. MIC for vancomycin should be performed in all laboratories to keep record of increasing resistance of enterococci to vancomycin and for early detection of vancomycin resistance by strain of enterococci.
